# Electrochemical Impedance Spectroscopy in the Characterisation and Application of Modified Electrodes for Electrochemical Sensors and Biosensors

**DOI:** 10.3390/molecules27051497

**Published:** 2022-02-23

**Authors:** Christopher M. A. Brett

**Affiliations:** Department of Chemistry, CEMMPRE, University of Coimbra, 3004-535 Coimbra, Portugal; cbrett@ci.uc.pt

**Keywords:** electrochemical impedance spectroscopy, modified electrodes, constant phase element, charge transfer resistance, Warburg impedance, sensors and biosensors

## Abstract

Electrochemical impedance spectroscopy is finding increasing use in electrochemical sensors and biosensors, both in their characterisation, including during successive phases of sensor construction, and in application as a quantitative determination technique. Much of the published work continues to make little use of all the information that can be furnished by full physical modelling and analysis of the impedance spectra, and thus does not throw more than a superficial light on the processes occurring. Analysis is often restricted to estimating values of charge transfer resistances without interpretation and ignoring other electrical equivalent circuit components. In this article, the important basics of electrochemical impedance for electrochemical sensors and biosensors are presented, focussing on the necessary electrical circuit elements. This is followed by examples of its use in characterisation and in electroanalytical applications, at the same time demonstrating how fuller use can be made of the information obtained from complete modelling and analysis of the data in the spectra, the values of the circuit components and their physical meaning. The future outlook for electrochemical impedance in the sensing field is discussed.

## 1. Introduction

The development of electrochemical sensors and biosensors requires three essential steps, namely preparation, characterisation and testing, for applications in synthetic and natural samples. Preparation and characterisation are intimately linked to each other since characterisation should be performed at various stages of sensor construction, when this involves various steps, for example after the addition of each modifier layer. Characterisation is performed by various techniques, both electrochemical and non-electrochemical. The non-electrochemical techniques are normally those of surface analysis, microscopic and spectroscopic. Of the former, the most use is made of atomic force microscopy and scanning electron microscopy, the surface analysis techniques that are used to examine the morphology of surface layers of materials in general. Electrochemical techniques are usually voltammetric, occasionally potentiometric, and impedance. Impedance spectroscopy has been used as a characterisation technique for materials for over a century. Its widespread use before the 1980s, just as was the case for electrochemical pulse techniques, was hindered by the practical difficulties in applying the potential waveform to the electrodes in an accurate way and being able to analyse the response in a short time period. Analysis of the impedance was reliant on the use of alternating current bridges, Lissajous figures and phase-sensitive detectors [1]. With the advent of transfer function analysers and powerful microprocessors, these difficulties were overcome, and it is now easy to record complete impedance spectra, usually switching from one frequency to the next automatically, the only limitation being the number of cycles needed to obtain “reliable” data at a particular frequency, with the minimum number varying according to the value determined by the instrument manufacturer. Thus, it is much easier nowadays to record and analyse full impedance spectra in several minutes, which is important for practical situations, in the sensor context for sensing or for characterising the stages of sensor build-up.

Electrochemical impedance spectroscopy (EIS) has been the subject of monographs and chapters in textbooks, e.g., [1,2,3,4,5,6], which present a wide variety of types of application. It can be used to investigate any system which exhibits interfacial electrical phenomena associated with chemical reactions, and the most common types of application at present are arguably evaluation of power sources (batteries, fuels cells, etc.) and corrosion phenomena.

After a brief description of the fundamentals of EIS which are necessary in order to be able to use the technique adequately for electrochemical sensor research, illustrative examples will be given, chosen to show what can be achieved by EIS as a characterisation technique and as a technique for quantitative determinations of electroactive species. Some of the more common ways of using the information from a full analysis of impedance experiments will be presented. Finally, the outlook and future challenges are considered.

## 2. Fundamentals of Electrochemical Impedance Spectroscopy

Electrochemical impedance spectroscopy relies on the measurement of the impedance characteristics of an electrical system at different frequencies of an applied perturbation, which is usually sinusoidal. Normally, and certainly in sensor applications, this sinusoidal voltage perturbation is small, of the order of 10 mV amplitude, in order to obtain a linear response, which simplifies the analysis of the spectra.

An electrode-solution interface can be modelled, at its simplest, by a capacitance, representing charge separation in the interfacial region, and a resistance, representing the resistance to charge (usually electron) movement across the interface. In the non-faradaic region of applied potential, where there is no charge transfer, just a capacitance suffices. These processes occur in parallel since where there is electron movement across the interface, there is no charge separation. The impedance of an electrode– or modified electrode–solution interface can be directly measured. Data from impedance experiments contain more information than does a voltammetric experiment, owing to, amongst other factors, the large range of timescales which is probed. This means that it is a more sensitive technique for probing changes to the electrode surface and the electrode–solution interface but, at the same time, this can make interpretation more difficult.

### 2.1. Basics of Electrochemical Impedance

In an electrochemical cell, there is separation of charge at the electrodes, there can be movement of charge or, at high frequency, induction due to the electric field. The electrical equivalent circuit can thus contain capacitors, resistors or inductors, respectively. Induction phenomena only need to be considered at very high frequency or in low-frequency relaxation phenomena, and will not be described here, as they are not relevant for electrochemical sensors. Additionally, owing to the way the electrochemical cell is physically made, and in the presence of a large quantity of electrolytes to carry the current and suppress charge migration effects, all important interfacial phenomena are concentrated close to the sensor electrode– or modified electrode–solution interface. In such cases, modelling of the impedance characteristics only needs to consider this interface, with the rest of the cell being represented by a cell resistance in series, which usually has a small value compared to the rest of the cell [1].

The measurement of electrochemical impedance normally involves applying a small sinusoidal voltage perturbation superimposed on a fixed value of applied potential at frequencies which typically vary from 100 kHz down to 10 mHz. The amplitude and change in phase of the current response to this perturbation of the applied potential is measured, from which the impedance can be deduced.

The excitation signal, expressed as a function of time, is normally written as:

(1) $$E_{t}=E_{0}\sin\left(\omega t\right)$$

where *E_t_* is the potential at time *t*, *E*_0_ is the amplitude of the signal and *ω* is the radial frequency. The radial frequency, *ω* (radians/second), is related to the frequency, *f* (in Hertz), by *ω* = 2π*f*.

In a linear system, the response signal, *I_t_*, is shifted in phase (*φ*) with respect to the voltage perturbation:

(2) $$I_{t}=I_{0}\sin\left(\omega t+\varphi \right)$$

The impedance is thus:

(3) $$Z_{t}=\frac{E_{t}}{I_{t}}=\frac{E_{0}\sin\left(\omega t\right)}{I_{0}\sin\left(\omega t+\varphi \right)}=Z_{0}\frac{\sin\left(\omega t\right)}{\sin\left(\omega t+\varphi \right)}$$

and is here expressed in terms of an impedance magnitude, *Z*_0_, and a phase shift, *φ*.

Considering the Euler formula (exp(*i*x) = cos(x) + *i*sin(x)) and that the impedance can be written as:

(4) $$Z_{t}=Z_{0}\left(cos\varphi +isin\varphi \right)$$

it is seen that the impedance can be divided into a real part and an imaginary part that can also be written as:

(5) $$Z_{t}=Z\prime +i\;Z\prime \prime$$

For sensor applications, the impedance data are usually presented in a complex plane plot, with the real part (*Z*′) plotted on the *x*-axis and the imaginary part (*Z*″) plotted on the *y*-axis, which is also referred to as a “Nyquist Plot”. The advantage of this representation is that the features of the impedance spectrum at lower frequencies are more prominent than those at higher frequencies (often the lower-frequency phenomena are the most important in sensors), while the disadvantage is that the frequency values are not explicitly shown. An alternative representation is of impedance magnitude and phase angle as a function of the logarithm of frequency, which shows all the information, but it is less easy to visualise the physical model of the system at low frequency. This Bode representation is most used in corrosion studies and in studies of materials.

In impedance experiments, different frequencies of the voltage perturbation are used to obtain the impedance response as a function of frequency, usually scanning logarithmically over a frequency range that is typically between 100 kHz and 0.01 Hz, with a specified number of frequencies per frequency decade (often 5 or 10), a frequency spectrum. For this reason, the technique is often known as electrochemical impedance spectroscopy (EIS). Normally, amplitudes of 5–10 mV sinusoidal perturbation ensure a linear response.

Linearity can be verified through the use of the Kramers–Kronig transforms, in which the imaginary part of the impedance, *Z*″, at any chosen frequency, can be calculated from the full frequency spectrum of the real part, *Z*′. Any differences between the calculated and experimental values of *Z*″ are evidence of non-linearity [1].

### 2.2. Physical Model of the Electrochemical Cell and Electrical Equivalent Circuits

As mentioned previously, conditions are usually created in electrochemical cells so that the observed response is due to processes occurring at the electrode– or modified electrode–solution interface. This is the case for the vast majority of voltammetric and impedance experiments. Electrical equivalent circuits are used to model the working electrode interfacial region and processes. The rest of the cell can then be represented by a cell resistance, *R*_Ω_.

The following three simple, but increasingly complex circuits, representing phenomena at an inert metallic electrode immersed in electrolyte solution with electroactive species, are all important for electrochemical sensing.

**Figure 1:**
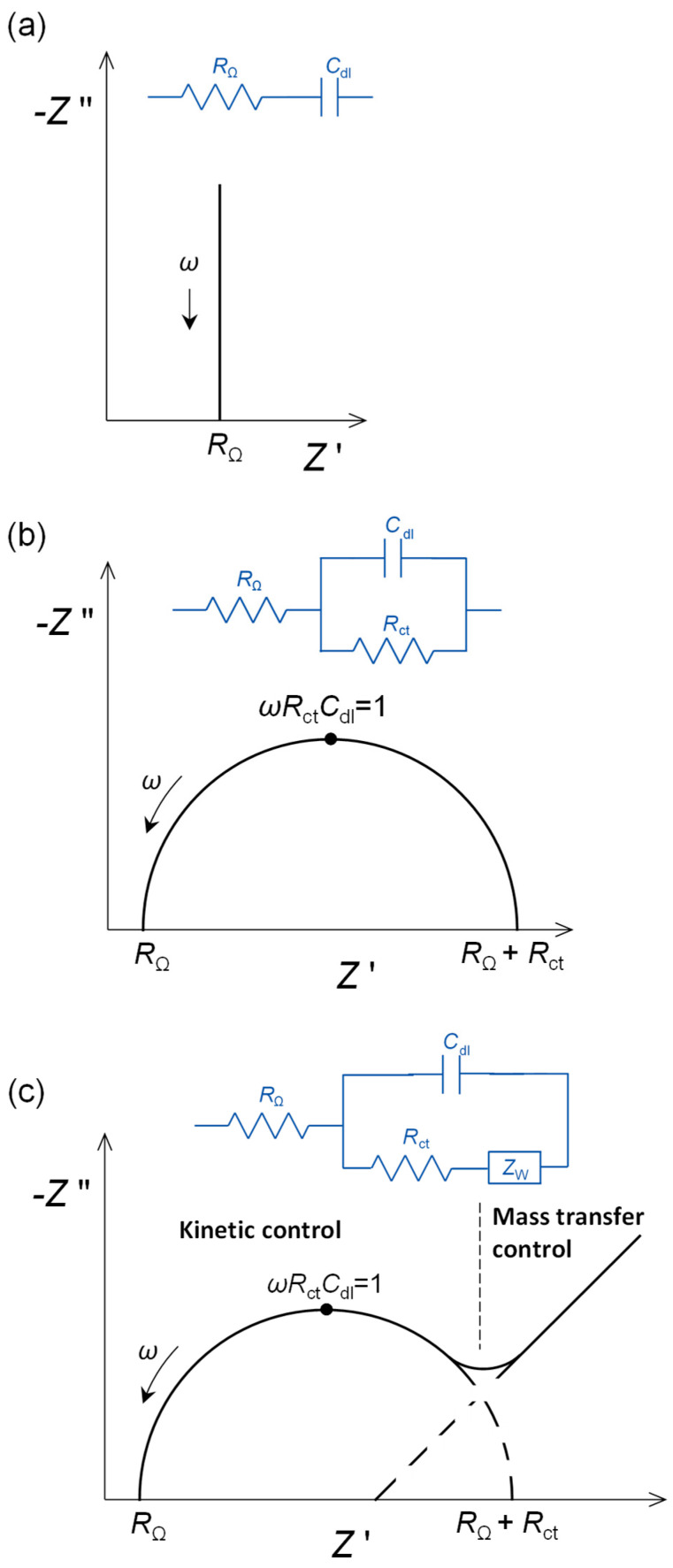
Complex plane impedance plots for selected electrical equivalent circuits: (**a**) capacitive interfacial response, (**b**) faradaic electron transfer reaction controlled by kinetics over the whole frequency range and (**c**) electron transfer reaction with mass transfer control at low frequencies. *R*_Ω_: cell resistance, *R*_ct_: electron transfer resistance, *C*_dl_: double-layer capacitance, *Z*_W_: Warburg impedance.

(a) At applied potentials where no electrode process occurs, there is ordering of the charge at the electrode interface on the solution side, often referred to as the double layer, and this charge will be compensated by sufficient charge polarisation within the electrode. The electrical equivalent circuit element is a capacitor, and in such a case, the full equivalent circuit is *R*_Ω_ in series with the double-layer capacitance, *C*_dl_. The impedance plot is a vertical straight line, as shown in Figure 1a, with the impedance components being given by:

*Z*′ = *R*_Ω_   *Z*″ = −1/(*ωC*_dl_) (6)

The value of the capacitance also depends on the area of the electrode, being larger the greater the area. Typical values would be 50–100 µF cm^−2^. This is sometimes referred to as non-faradaic impedance.

(b) If an electrode process occurs at a particular applied potential, that is controlled by the rate of charge transfer across the electrode–solution interface over the whole frequency range, then the charge transfer is represented by a charge transfer resistance, *R*_ct_. The resistance, *R*_ct_, is placed in parallel with the double-layer capacity, *C*_dl_, since charge transfer and charge separation happen at different sites on the electrode surface. A complex plane plot of the impedance affords a spectrum with a semicircle of diameter *R*_ct_, see Figure 1b.

(7) $$Z^{\prime }=R_{\Omega }+\frac{R_{\mathrm{ct}}}{1+{\left(\omega R_{\mathrm{ct}}C_{\mathrm{dl}}\right)}^{2}}\;Z\prime \prime \;=-\frac{R_{\mathrm{ct}}^{2}C_{\mathrm{dl}}}{1+{\left(\omega R_{\mathrm{ct}}C_{\mathrm{dl}}\right)}^{2}}$$

(c) The maximum of the semicircle corresponds to *ωR*_ct_*C*_dl_ = 1. The value of *R*_ct_ depends on the rate of charge transfer, analyte concentration and the electroactive area of the electrode, becoming smaller in value the larger the area. The normalised values of resistances are expressed in Ω cm^2^. Modification of an electrode, for example by a nanomaterial, may lead to a large increase in electroactive area and, owing to this, *R*_ct_ becomes smaller. In such a case, it cannot be deduced that the electron transfer rate has increased due to incorporation of the nanomaterial, without first compensating for the increase in electroactive surface area.

The situation can occur where there is kinetic control at high frequencies, but owing to the longer timescale, at lower frequencies the charge transfer rate is controlled by diffusion of species in solution. Diffusion control is represented by a Warburg impedance, *Z*_W_, and corresponds to a line at phase angle 45° in the complex plane plots. The generic shape of the corresponding complex plane spectrum is shown in Figure 1c, which is often found in sensor applications, for example when model electroactive species are used to probe to what extent a modifier film, that blocks electron transfer, covers the electrode surface.

More complex equivalent circuits often need to be invoked for modified electrodes, as will be seen in some examples below. For example, the modifier film of a film-modified electrode can itself have a resistance and charge separation that could be represented by a second *RC* parallel combination in series with the circuits above.

**Figure 2:**
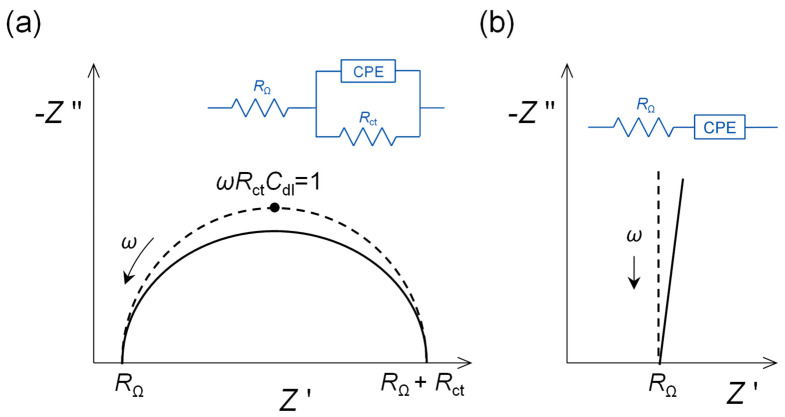
Complex plane impedance plots for selected electrical equivalent circuits with ideal capacitor response (dotted lines) replaced by non-ideal capacitor constant phase elements (CPE) (solid lines). (**a**) Faradaic electron transfer reaction controlled by kinetics over the whole frequency range, and (**b**) capacitive interfacial response.

In practical sensing situations, the complex plane plots, even with model electroactive species, do not usually afford perfect semicircles. The semicircles are “depressed” (Figure 2a), and the expected vertical straight lines for capacitors appear at angles of less than 90° (Figure 2b). The reason for this can be attributed to surface non-uniformity and roughness, and even porosity, in that each local sub-microscopic area gives rise to its own *RC* combination, but what we observe macroscopically is the sum of all of these contributions. This is often described as frequency dispersion and can be taken into account in the mathematical equations describing the physical model by using a constant phase element (CPE). For a non-ideal capacitor, this has the form:

CPE = −1/(*iωC*)*^α^* (8)

where *α* is called the CPE exponent, that varies between 1.0 for a pure capacitor and 0.5 for a porous electrode [1]. Values of 1.0 are, in practice, uncommon. For example, a bare, polished glassy carbon electrode (GCE) in the capacitive region often affords a CPE exponent of between 0.85 and 0.90.

### 2.3. Analysis of Experimental Impedance Spectra

Experimental impedance data are analysed by fitting them to an appropriate electrical equivalent circuit. It is very important that a physical model of the electrode– or modified electrode–solution interface and electrode process that can be justified is first developed. Fitting of all circuit parameters can then be performed using software that is usually supplied with the electrochemical impedance instrumentation, starting the fitting with reasonable initial values of the circuit parameters. Some of the more-simple circuits are often incorporated in the instrument software and that can be used as a first step, referred to as “instant fitting”. Fitting is normally carried out by the complex, non-linear least squares method in order to minimise the errors between fitted and experimental impedance values. Such errors are usually specified next to the optimised values of the circuit parameters and should be carefully monitored. Large errors generally mean that either the experiment was not properly carried out, or that the electrical equivalent circuit is not appropriate. Nevertheless, adding circuit elements to give a better fit without physical justification for their existence must be avoided. Big error values can also occur when only a small part of a feature is present in the spectrum, so that the error is expressing the inherent uncertainty in the fitting. This happens, for example, when the beginning of a very large-diameter semicircle appears at the low-frequency end of the spectrum.

Details on the representation and analysis of impedance spectra for more complex situations can be found in [2,3]. Some of them will be seen in the examples below.

## 3. EIS as a Sensor Characterisation Technique

Important sensor characterisation experiments have been performed in many studies, and characterisation continues to be the principal use of EIS in a sensor context [7]. EIS experiments can be carried out during the build-up of layers in layer-by-layer sensor platforms and can give added information on what is occurring, and also probe permanent differences after construction. Some illustrative examples will be given in the following sections.

### 3.1. Self-Assembled Bilayer Structures

Self-assembled structures are discussed first because they can be easily probed by EIS without a redox probe marker. There are many examples in the literature, see [8], and this represents an interesting pathway for sensor development in the future since self-assembly has a number of advantages, such as that only small quantities of the chemical reagents are needed.

The first example involves the spontaneous self-assembly of alkanethiols on gold electrode surfaces in the absence of any Faradaic reactions, and without any redox probe [9].

The impedance of a bare polished and conditioned polycrystalline gold electrode in the non-Faradaic region would be expected to be that of a pure capacitor, as in Figure 1a. However, the phase angle is less than 90° and can be represented by a non-ideal capacitor CPE (see Figure 2b), with an α exponent of 0.85. After self-assembly of the alkanethiol, that occurs easily because of the strength of the gold–sulphur bonds, the exponent increases to 1.0, a pure capacitor. This means that the surface is now completely smooth and uniform at the molecular/nanometric level.

**Figure 3:**
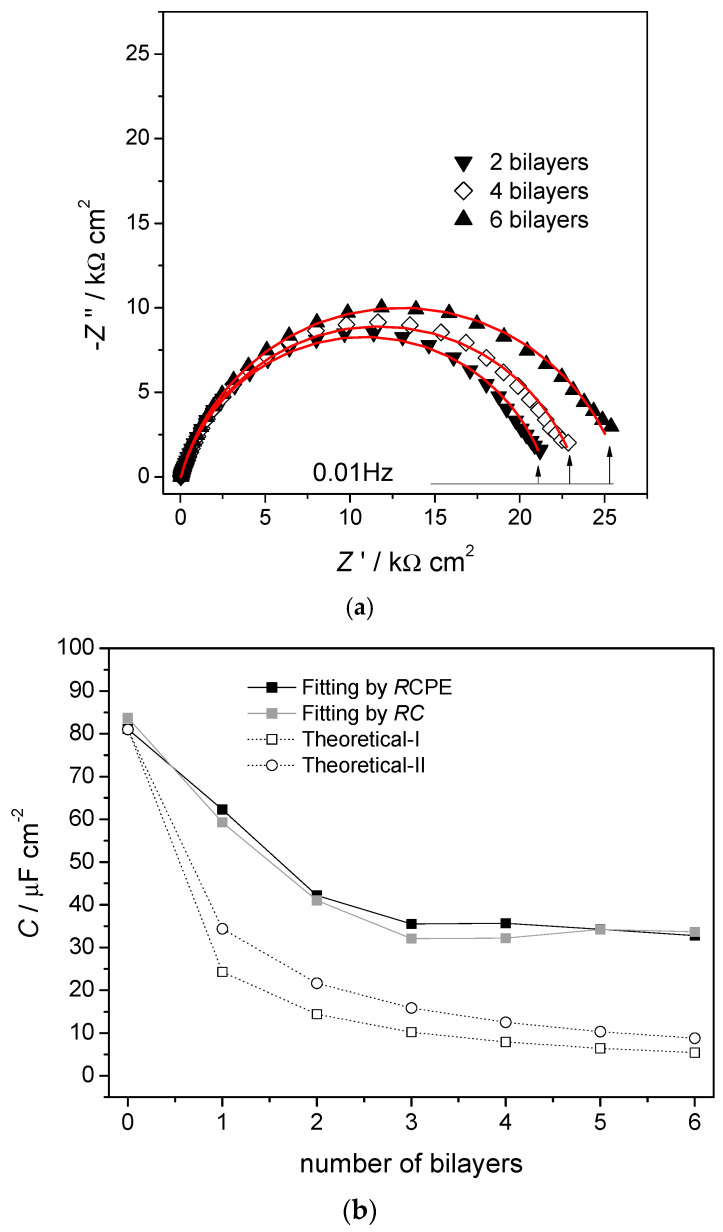
(**a**) Complex plane impedance plots recorded at gold quartz crystal microbalance (AuQCM)-modified electrodes in 0.05 M acetate +0.1 M KBr buffer solution, pH 5.0, at the OCP. (**a**) AuQCMMPS(−)/PDDA(+)/{HA/Mb}_2–6_, lines show equivalent circuit fitting to the circuit in Figure 2b. (**b**) Capacitance values. Dark squares, from *R*CPE equivalent circuit fitting to the experimental spectra; light squares, from *RC* semicircle modelling; (0) theoretical-I *C*_i_ = 34 μF cm^−2^ and (O) theoretical-II, *C*_i_ = 59 μF cm^−2^ obtained by summing contributions from successive bilayers. From [10], reprinted with the permission of the American Chemical Society, Washington, DC, USA.

A more complex example involves the spontaneous build-up of bilayers of myoglobin (Mb) and hyaluronic acid (HA) on a gold quartz crystal microbalance (AuQCM) electrode previously functionalised with sodium 3-mercapto-1-propanesulfonate (MPS) and poly (diallyldimethyl-ammonium chloride) (PDDA) [10]. Impedance spectra after the adsorption of each bilayer are shown in Figure 3a. The electrical equivalent circuit is the one shown in Figure 2a, but it is not immediately obvious what information can be deduced from the experimental spectra except for the values of *R*. Analysis of the capacitance, either ideal or non-ideal as a CPE, provides further information, taking each bilayer as having its own value of capacitance and summing the values of *C*^−1^ corresponding to capacitances in series. This analysis shows that for up to three bilayers, the capacitance values vary with each bilayer, but they become constant above three bilayers. Simple theoretical models employing constant bilayer capacitance values do not provide the correct profile, see Figure 3b. These deductions are corroborated by information from quartz crystal microbalance experiments and atomic force microscopy. Further details may be found in [10], and this illustrates the value of doing a full analysis of the spectra, and not just of *R,* which is common practice in many published articles.

### 3.2. Layer-by-Layer Modified Electrode Structures

Differences between layer-by-layer structures can be conveniently illustrated by recent papers that concern GCE modified by nanomaterials, without the addition of a redox probe.

**Figure 4:**
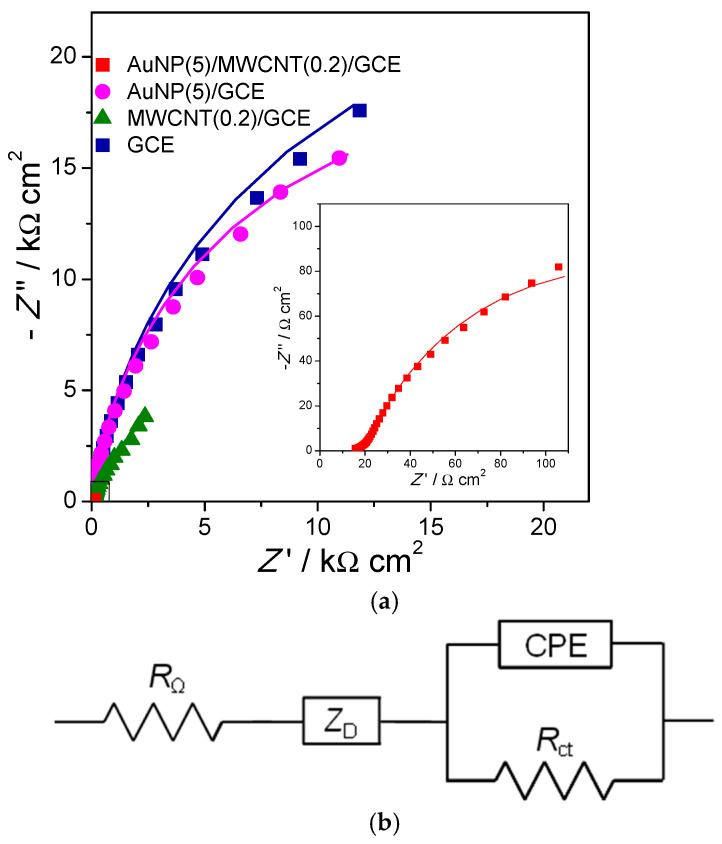
(**a**) Complex plane impedance plots at different electrodes in BR buffer (pH = 6) containing 9.0 mM BPA. Inset is the magnified plot of AuNP (5)/MWCNT (0.2)/GCE. Lines show equivalent circuit fitting. (**b**) Electrical equivalent circuit used to fit the spectra. From [11], reprinted with the permission of Elsevier, Amsterdam, The Netherlands.

First, GCE are modified with multiwalled carbon nanotubes (MWCNT) in chitosan, and then gold nanoparticles (AuNP) are deposited and they decorate the MWCNT, with the modified electrode finally being used to detect and quantify bisphenol-A (BPA) [11]. Impedance spectra can be recorded at different stages in the build-up process of the sensor and are shown in Figure 4 together with the equivalent electrical circuit used to model the spectra. The circuit includes two features, namely a high-frequency diffusion impedance representing diffusion through the modifier layers and a parallel *R*CPE combination to model the modified electrode–solution interface. For bare and AuNP-modified electrodes, it was not necessary to include the diffusion impedance for good fitting.

**Figure 5:**
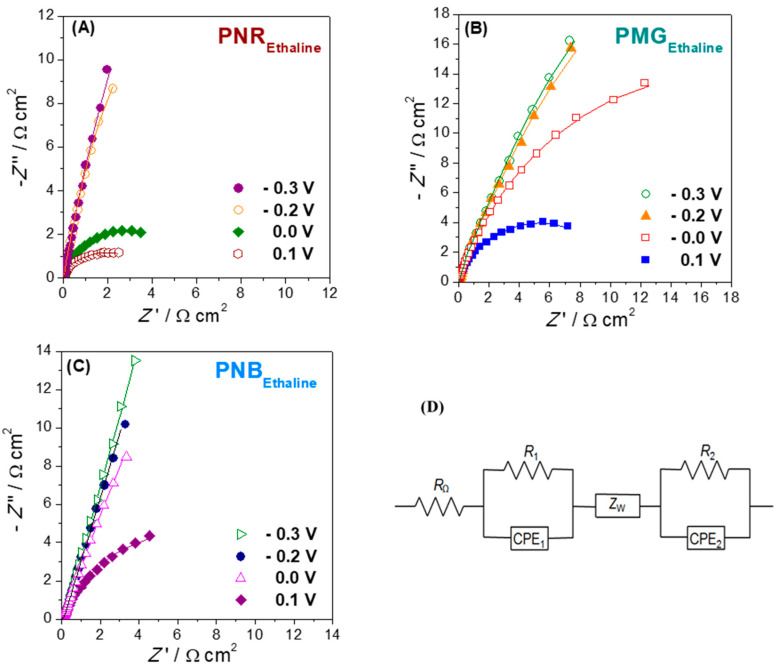
Complex plane impedance plots recorded in 0.1 M BR (pH 3.0) buffer solution at −0.3, −0.2, 0.0 and 0.1 V vs. Ag/AgCl at (**A**) PNR_Ethaline_/Fe_2_O_3_NP/GCE, (**B**) PMG_Ethaline_/Fe_2_O_3_NP/GCE and (**C**) PNB_Ethaline_/Fe_2_O_3_NP/GCE. (**D**) Electrical equivalent circuit used to fit the spectra. From [12], reprinted with the permission of Elsevier.

The second illustration concerns electrodes modified with iron oxide nanoparticles (Fe_2_O_3_NP) in a chitosan dispersion (rather than MWCNT in chitosan), deposited on GCE [12]. On top of this, a polyphenazine redox polymer film is grown by potential cycling electropolymerisation. Three different polyphenazine films were grown in sulfuric acid-doped ethaline (choline chloride and ethylene glycol in a 1:2 ratio) deep eutectic solvent (DES). These were poly (neutral red) (PNR), poly (methylene green) (PMG) and poly (Nile blue) (PNB). The impedance spectra recorded afterwards in Britton–Robinson aqueous buffer solution showed the differences between the electrical characteristics of the three polymers, which can be ascribed to the different monomer structures. The variation with applied potential, another important variable, is similar for all three polymers. These impedance spectra show three features, each in a different frequency range. Apart from the two features shown in Figure 4 for electrodes modified with MWCNT and AuNP (that are diffusion impedance for diffusion through the modifier film in series with a parallel *R*CPE representing the electrode–solution interface), to model the spectra at very high frequencies, an additional parallel *R*CPE combination is needed, and that represents the interface between the electrode support and the modifier film, see Figure 5.

The general circuit shown in Figure 5 can be successfully used to model other cases involving nanomaterial-modified electrodes on which an electroactive redox polymer film is deposited.

An example in which the redox probe is used for characterisation during the build-up of the sensor is found in [13]. The redox probe is used to probe the effectiveness of immobilisation of successive elements of an immunosensor, although the quantification itself is performed by cyclic voltammetry, obtaining a detection limit of 0.8 ng/mL for carcinoembryonic antigen detection. Other examples are described in [14], in the assembly of aptasensors with carbon nanomaterials. Aptamers are DNA-derived oligonucleotides and an alternative to native antibodies and can be tuned more easily to the structure of specific analyte species.

## 4. EIS as a Sensing Technique

EIS can be used as a diagnostic or quantitative sensing technique. For this purpose, either the impedance response to a redox probe needs to change when the (modified) electrode is exposed to an analyte or the electrical characteristics of the (modified) electrode itself change. Both of these have been described, but the former is the most used in reports in the literature, which will be exemplified below.

### 4.1. EIS Using a Redox Probe

Redox probes should exhibit fast kinetics, and normally not interfere with the interfacial properties of the modified electrode. There is thus a tendency to employ the same model redox couples which are used to characterise electrode materials and modified electrodes, mainly the hexacyanoferrate (III)/(II) redox couple and the hexaammineruthenium (III)/(II) redox couple. The use of equimolar mixtures of hexacyanoferrate (II) and hexacyanoferrate (III) is particularly prevalent, with typical concentrations being around 1 mM.

The use of equimolar concentrations fixes the open-circuit potential (OCP) at the formal potential, which is usually around 0.18 V vs. Ag/AgCl, varying a little depending on the reference electrode and associated electrolyte concentration. The OCP is measured at the beginning of the experiment by the instrument and this value is then applied by the potentiostat, with such large concentrations of the redox probe that the OCP value does not vary during recording of the spectrum. These couples are used because the electrode reactions usually have fast kinetics and thus show an impedance profile of the type shown in Figure 1c. Of the two redox probes mentioned, the latter is better since it undergoes an outer-sphere electron transfer reaction. However, most of the literature reports use hexacyanoferrate (III)/II). In some reports, it appears so evident to the authors that a redox probe is needed that, unfortunately, they neglect to mention this in the text and figure captions.

The fact that both hexacyanoferrate (III)/II) and hexaammineruthenium (III)/(II) couples involve complex ions with a charge, negative and positive, respectively, may differently influence the access of these species (through pores, or near the edge of uncovered areas, of a blocking modifier layer) to the electrode substrate underneath. This could be important if the redox couple is being used to probe the degree of coverage as a function of analyte concentration. It has been suggested that, at least on gold electrodes, the hexaammineruthenium (III)/(II) redox couple may have an added advantage in that hexacyanoferrate can cause corrosion of the gold electrode over time [15], but its use is not widespread.

**Figure 6:**
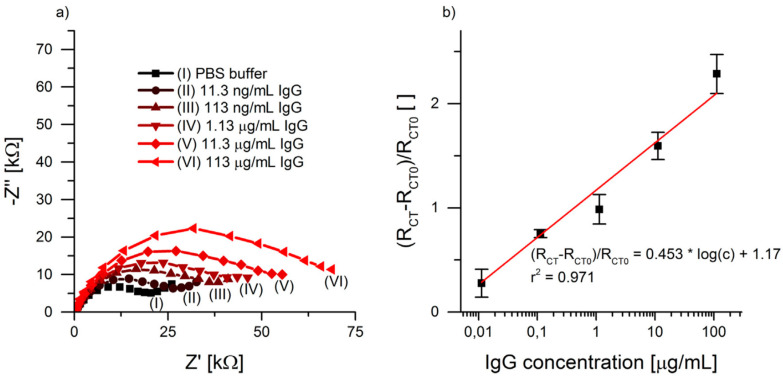
(**a**) Complex plane plots of screen-printed biosensors with MUA/IgG-Ab/BSA functionalisation after 30 min of incubation in (I) PBS, (II) 11.3 ng/mL IgG, (III) 113 ng/mL IgG, (IV) 1.13 μg/mL IgG, (V) 11.3 μg/mL IgG and (VI) 113 μg/mL IgG. (**b**) Calibration curve of buffered IgG solutions with relative *R*_CT_ vs. IgG concentration in half-logarithmic form, number of measurements = 3. MUA: 11-mercapto undecanoic acid, IgG: immunoglobulin G antibody, BSA: bovine serum albumin, PBS: phosphate-buffered saline. From [15], reprinted with the permission of Elsevier.

Analysis of the impedance spectra should be to the electrical equivalent circuit shown in Figure 1c, with a CPE replacing the double-layer ideal capacitance, *C*_dl_. In published articles, often only the value of the charge transfer resistance associated with the redox couple is determined, together with its variation with time or under different experimental conditions. For example, if there is blocking of adsorption, then the value of *R*_ct_ will increase, since the area where the electrode reaction can occur will be smaller. Thus, it can be used as a tool for the measurement of surface coverage, as well as for concentration dependence, as seen with the biosensor shown in Figure 6. Many recent examples are provided in the reviews in [16,17], which focus on medical diagnostics. The importance of nanocomposite transducers and their evaluation and application employing redox probes is highlighted in [18], which also considers non-faradaic impedance sensing (see Section 4.2).

More recently, as illustrated in [19], the hexacyanoferrate redox couple was used to characterise an impedimetric sensor with direct electron transfer-type glucose dehydrogenase (GDH). EIS was then used directly as a measurement technique in a glucose biosensor with FAD-dependent GDH immobilised on an alkanethiol-modified gold electrode, which showed a direct glucose concentration dependence of the change in 1/*R*_ct_, with values deduced from fitting of the impedance spectra. It was also shown that calibration plots can be made using just one single frequency, in this case 5 MHz, which considerably simplifies and increases the speed of analyte determination. This proof-of-concept paves the way for similar developments in the future.

As an example of EIS application to DNA sensing with a redox probe, a ZnO-nanorod-based DNA biosensor was developed to detect complementary target single-stranded DNA (ssDNA) of the BCR/ABL fusion gene [20]. Electrochemical impedance spectroscopy, in the presence of 1.0 mM [Fe(CN)_6_]^3−/4−^, was used to study the immobilisation and hybridisation of ssDNA at the electrode surface. The complementary target ssDNA of BCR/ABL could be quantified in the concentration range from 1.0 pM/L to 1.0 nM/L, based on the dependence of the change in *R*_ct_ on the logarithm of its concentration.

An impedimetric detection system for the DNA–ligand interaction measured the increase of charge transfer resistance of [Fe(CN)_6_]^3−/4−^ after hybridisation of the complementary target with increasing amounts of capture probe [21].

**Figure 7:**
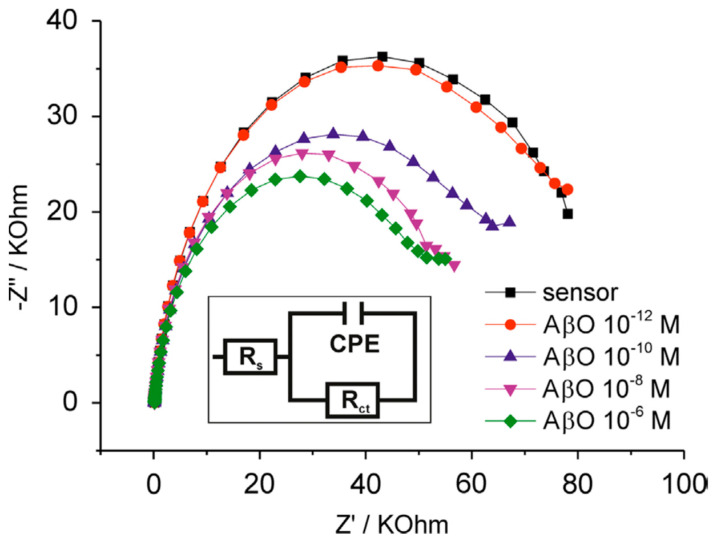
Biosensor response to Aβ oligomers as measured by EIS. The response of the biosensors to AβO was analysed by EIS. Following equilibration in vector solution alone (sensor), cumulative additions of AβO (10^−12^−10^−6^ M total Aβ peptide concentration) were performed for 20 min each prior to rinsing and EIS measurement in a solution of PBS containing 10 mM Fe(CN)_6_^3−^/^4−^ (aq). Inset shows the Randles equivalent circuit model for this system, where *R*_s_ = solution resistance, *R*_ct_ = charge transfer resistance and CPE = constant phase element, a model of an imperfect double-layer capacitor. From [22], reprinted with the permission of Elsevier.

Figure 7 shows an example which makes clear the difference between “label-free” and the use of a redox probe [22]. The change in charge transfer resistance for the hexacyanoferrate redox couple associated with a biosensor platform built on gold screen-printed electrodes for the detection of Alzheimer’s amyloid-beta oligomers forms the basis of the detection strategy. EIS was also used for characterising the modified electrode at different stages of the platform construction [22].

**Figure 8:**
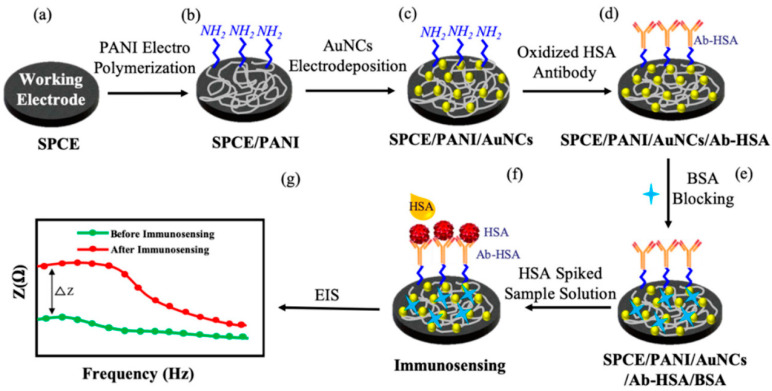
Schematic of the systematic protocol for SPCE surface modification and immunosensing. (PANI—polyaniline, AuNCs—gold nanocrystals, HSA—human serum albumin, Ab-HSA—anti-human serum albumin antibody, BSA—bovine serum albumin, EIS—electrochemical impedance spectroscopy). From [24], reprinted with the permission of MDPI.

Impedance immunosensors, where EIS is used to directly determine biomolecular recognition events as well as the potential advantages of both screening and online monitoring without being destructive, are pointed out in [23]. Conducting polymers can be used to immobilise the antigens, and Figure 8 illustrates the basic concept [24].

Impedimetric biosensors with redox probes have found various applications in forensic science [25]. They have also been used in modified electrode sensors with conducting polymers such as polypyrrole [26].

EIS emerges as a promising technique for quantifying bacteria in waters using molecularly imprinted sensors, with a view to ensuring clean and safe water [27]. Novel chitosan-Nafion composites for the fabrication of highly sensitive impedimetric and colorimetric As (III) aptasensors have been prepared, in the case of impedimetric sensing based on the change in charge transfer resistance of the hexacyanoferrate couple with increasing sub-micromolar As (III) concentration, affording sub-nanomolar detection limits [28].

### 4.2. EIS without a Redox Probe

**Figure 9:**
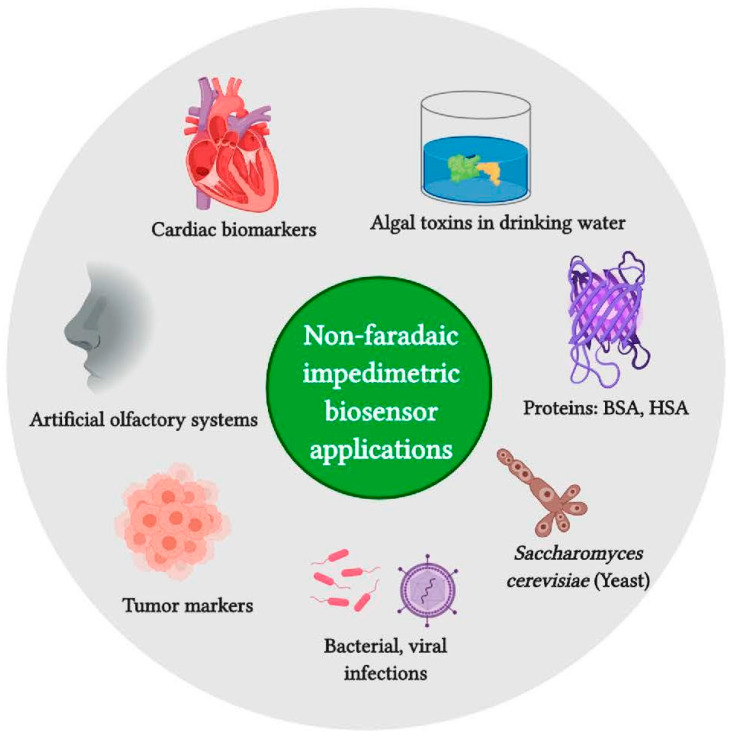
Application areas for non-faradaic impedimetric biosensors. BSA: bovine serum albumin, HSA: human serum albumin. From [29], reprinted with the permission of Elsevier.

EIS without a redox couple as a probe is often referred to as non-faradaic sensing, although this can be a misnomer if impedance spectra are recorded at a potential at which oxidation or reduction of a component of the redox system being investigated occurs. The range of biological applications of non-faradaic sensing [29] can be seen in Figure 9.

The values of impedance can be used to probe changes in conductivity. For example, a L-lactate selective impedimetric biosensor based on a lactate dehydrogenase (LDH)/pyruvate oxidase (PyrOx) bio-selective membrane on a screen-printed carbon electrode measured the change in impedance caused by the generation of charged ions (CH_3_COO^−^, H^+^ and HCO_3_^−^), from PyrOx catalysed pyruvate oxidation, the pyruvate resulting from L-lactate oxidation. Equivalent circuit deduction of the values of resistance at the interface between the two enzyme layers, *R*_IM_, and the corresponding parallel capacitance, represented as CPE_IM_, enabled the construction of linear calibration plots for L-lactate, with the detection limits being 17 and 20 µM, for *R*_IM_ and CPE_IM_, respectively. Further details can be found in [30].

In [31], nano-ZnO/CuO and nano-ZnO nitrocellulose membrane biosensors, fabricated using a simple and inexpensive sonication technique, were investigated and compared by EIS. Changes in impedance phase angles, at a frequency of 100 Hz, were used to establish dose-dependent responses for C-reactive protein. A similar sort of approach was used in [32] for identifying antigen–antibody binding interactions using a non-faradaic impedimetric sensor fabricated on a printed circuit board (PCB) chip.

The value of *R*_ct_ from impedance spectra is sometimes used to calculate an apparent rate constant, *k*_app_, of the electrode reaction according to the equation:

*k*_app_ = *RT*/((*nF*)^2^*AR*_ct_*c*) (9)

where *A* is the electrode area, *c* is the bulk concentration of the electroactive species and all other parameters have their usual meanings. The obtained value needs to be interpreted with care because a large increase in the apparent rate constant is very probably simply due to a large decrease in exposed area (as seen with redox probes). For example, if 90% of the surface is inactive, the apparent rate constant would increase by a factor of 10. Thus, an alternative means should be used to calculate the standard rate constant, for example peak separation in cyclic voltammetry.

An interesting methodology was used to calculate the rate constant of oxidation of nucleic acid bases at a GCE subject to adsorption [33]. Low-power ultrasound at 20 kHz from an ultrasonic probe was used to keep the electrode surface clean during the recording of the impedance spectrum. The ultrasound does not affect the recording of the impedance since only one frequency is being sampled at a time. The standard rate constant, *k*_0_ (rather than the apparent rate constant), for the oxidation of the purine bases guanine, adenine and guanosine, was then determined without blocking effects using the equation:

*k*_0_ = *RT*/((*nF*)^2^*AR*_ct_*c*) (10)

in which *n* = 2. The ultrasound ensures that the calculation of *k*_0_ is not affected by any decrease in electrode surface area.

This strategy should be applicable to other compounds that are prone to adsorption on electrode surfaces and it is desired to ascertain the rate of oxidation or reduction of the species in solution.

**Figure 10:**
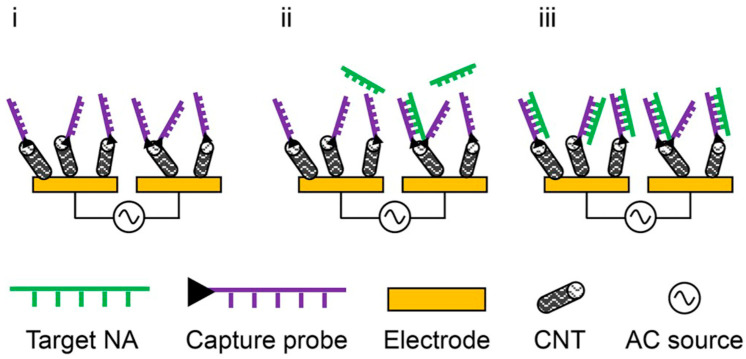
Heterogeneous, label-free, reagent-less sensing principle. Cubed laboratories use interdigitated electrodes and functionalised CNT for nucleic acid (NA) detection. Initially, a baseline impedance measurement was performed (**i**). Then, the single-stranded product of an asymmetric PCR mixed with hybridisation buffer was hybridised to the CNT-bound capture probes (**ii**). After washing with measurement buffer, another impedance measurement was performed (**iii**). All steps were performed while applying an AC field for di-electrophoresis, which supports specific hybridisation. From [34], reprinted with the permission of Elsevier.

Electrochemical DNA sensing, its principles and applications have been reviewed in [34], which illustrates the heterogeneous, label-free, reagent-less sensing principle for hybridisation detection, see Figure 10.

Another example of impedimetric sensing of DNA hybridisation is described in [35]; however, the authors have used a different approach with AC amplitudes of 1 V and very high frequencies of the order of 1 MHz, examining the transition between kinetic and diffusion control (see Figure 1c). Whilst this leads to analysable experimental results, it is evident that systems of this kind with such large sinusoidal voltage perturbations do not obey the linearity criteria that were discussed earlier in this article and the circuit could suffer from power losses. It is recommended that large sinusoidal voltage perturbations (i.e., more than 10 mV) should be avoided unless there is a very good reason for doing so.

Recently, there has been interest in coupling room temperature ionic liquids with non-faradaic impedimetric sensing. The RTILs are used for increasing the sensitivity of the measurements. Further details can be found in [29].

## 5. Future Outlook

Electrochemical impedance spectroscopy is a valuable electrochemical technique for characterisation of electrochemical systems and materials, which includes the characterisation of electrodes and modified electrodes. Some resistance to its use, as recently explicitly pointed out [36], is probably due mainly to a lack of knowledge of the basic principles of ac electrical circuits, that are used to represent the charge separation and charge transfer phenomena in the interfacial region on electrode surfaces. This is one of the main challenges for more widespread use of EIS in the future.

It is easy, nowadays, to record impedance spectra with modern electrochemical instrumentation using the default experimental conditions, with little questioning of whether the experimental conditions need to be adapted to the system under study. There is the necessity of procuring or devising an appropriate and adequate physical model to be represented by the electrical equivalent circuit and carrying out the fitting of the experimental spectra to it. This is paramount and especially important with sensors and biosensors, application to which is not as easy as in some other areas of electrochemistry. Additionally, care must be taken to carry out the recording of spectra such that they are fully reproducible. The reason can be traced to the fact that the impedance is very sensitive and is probing a large range of timescales, as is evident from the range of ac frequencies normally applied. This means that the spectrum will reflect tiny as well as large changes in the modified electrode, which is both an advantage and a possible disadvantage to quantitative sensing.

The recent literature demonstrates that electrochemical impedance spectroscopy has increasing application in the characterisation and application of electrochemical sensors and biosensors. This trend is expected to continue, at the same time with greater use of all the information that is supplied by full analysis of the impedance spectra.
